# High BMPR2 expression leads to enhanced SMAD1/5/8 signalling and GDF6 responsiveness in human adipose-derived stem cells: implications for stem cell therapies for intervertebral disc degeneration

**DOI:** 10.1177/2041731420919334

**Published:** 2020-05-18

**Authors:** Tom Hodgkinson, Francis Wignall, Judith A Hoyland, Stephen M Richardson

**Affiliations:** 1 Division of Cell Matrix Biology and Regenerative Medicine, School of Biological Sciences, Faculty of Biology, Medicine and Health, University of Manchester, Manchester Academic Health Science Centre, Oxford Road, Manchester, UK; 2 NIHR Manchester Musculoskeletal Biomedical Research Unit, Central Manchester Foundation Trust, Manchester Academic Health Science Centre, Manchester, UK

**Keywords:** Intervertebral disc degeneration, growth differentiation factor 6, mesenchymal stem cells, adipose-derived stem cells, nucleus pulposus, bone morphogenetic protein receptor, SMAD1/5/8, ERK1/2

## Abstract

Stem cell–based regenerative strategies are promising for intervertebral disc degeneration. Stimulation of bone-marrow- and adipose-derived multipotent stem cells with recombinant human growth differentiation factor 6 (rhGDF6) promotes anabolic nucleus pulposus like phenotypes. In comparison to mesenchymal stem cells, adipose-derived multipotent stem cells exhibit greater NP-marker gene expression and proteoglycan-rich matrix production. To understand these response differences, we investigated bone morphogenetic protein receptor profiles in donor-matched human mesenchymal stem cells and adipose-derived multipotent stem cells, determined differences in rhGDF6 signalling and their importance in NP-like differentiation between cell populations. Bone morphogenetic protein receptor expression in mesenchymal stem cells and adipose-derived multipotent stem cells revealed elevated and less variable expression of BMPR2 in adipose-derived multipotent stem cells, which corresponded with increased downstream pathway activation (SMAD1/5/8, ERK1/2). Inhibitor studies demonstrated SMAD1/5/8 signalling was required for rhGDF6-induced nucleus-pulposus-like adipose-derived multipotent stem cell differentiation, while ERK1/2 contributed significantly to critical nucleus pulposus gene expression, aggrecan and type II collagen production. These data inform cell regenerative therapeutic choices for intervertebral disc degeneration regeneration and identify further potential optimisation targets.

## Background

Intervertebral disc (IVD) degeneration is a leading cause of low back pain, for which at present, few effective management options are available. Preventative conservative treatments often offer short-term relief from symptomatic pain and improve functionality but fail to address the underlying progressive nature of disc degeneration. In cases of long-term and severe low back pain, costly and invasive surgical interventions, such as vertebral fusions or discectomies, are used as last-resort strategies. Outcomes from such procedures, though often positive, can result in altered spinal loading and degeneration of IVDs adjacent to the intervention site.^1,2^ Due to the failure of these conventional therapies to address the underlying pathology, there is a need to develop effective regenerative strategies targeting IVD degeneration. In particular, effective restoration of a healthy and functional extracellular matrix (ECM) in the central portion of the IVD, the nucleus pulposus (NP), has been a focus of research in the field.^3-5^ Several regenerative medicine strategies have been proposed, but the location and enclosed-nature of the NP within the IVD make adult stem cell therapy alongside small molecule biologics an attractive option.^5,6^

To maximise the efficacy of such therapies for IVD regeneration it is essential to select both the correct cell population and the correct instructive factor to ensure adoption of a healthy NP cell phenotype and stimulate appropriate ECM synthesis. Multipotent stem cells are found in numerous adult tissues including bone marrow (bone marrow–derived mesenchymal stem cells [MSCs]) and adipose tissue (adipose-derived stem cells [ASCs]). Cells from these sources show promise for IVD regeneration in preclinical models, where MSC implantation into degenerate IVDs increased disc height, upregulated ECM production and increased the expression of healthy-NP genes^5^ (and the references therein).

To control implanted and endogenous cell behavior, members of the transforming growth factor/bone morphogenetic protein (TGFβ/BMP) superfamily have been the most widely investigated. TGF- and BMP-family members are known to signal through different branches of the SMAD signalling pathway (SMAD2/3 and SMAD1/5/,8 respectively), which results in differential activation of down-stream transcription factors and phenotypic responses.^7^ Furthermore, the effect of each factor may well be cell-population dependent, making it necessary to develop a detailed understanding of the mechanisms behind differentiation to inform therapy decisions. Recently, members of the growth differentiation factor (GDF) sub-group of the BMP family, GDF5 and GDF6, have been shown to be promising candidates as biological therapies for cartilage and IVD regeneration.^8,9^ Work from our laboratory and others has demonstrated that stimulation of MSCs and ASCs with recombinant human (rh)GDF6 promotes their differentiation to an NP-like phenotype.^10-13^ Furthermore, rhGDF6 induces greater increases in NP-marker genes and proteoglycan production than other candidate growth factors, namely rhGDF5 and rhTGFβ3, particularly in ASCs.^10^

Reported differences in the responses of MSCs and ASCs to these BMP-family factors, including rhGDF6,^10^ and previous evidence of stem cell population-dependent chondrogenic potential.^14-18^ led us to hypothesize that inherent differences in signalling pathway activation and signal transduction existed between the populations. Importantly, donor-matched MSCs and ASCs showed different responses to rhGDF6, with ASCs differentiating to a phenotype more closely resembling NP cells and producing an ECM more closely resembling NP tissue.^10^

To maximise the efficacy of IVD regenerative cell therapies, it is critical to understand these different responses in mechanistic detail in order to select the most efficacious cell population partnered with the correct instructive bioactive factor. Therefore, the aim of this study was to identify and determine the relative importance of the signalling mechanisms by which ASCs respond differently than MSCs to perhaps the most promising discogenic factor identified to date, rhGDF6. To inform both therapeutic cell population source choices and identify important signalling mechanisms for potential future therapeutic manipulation, this study aimed to investigate rhGDF6 signalling in donor-matched MSCs and ASCs and gain insight into the mechanism of rhGDF6 induction of NP-like phenotypes in ASCs through examination of signalling pathway activation and pathway-specific blocking.

## Methods

### Extraction and culture of MSCs and ASCs

All procedures and experiments were performed with relevant NHS Health Research Authority National Research Ethics Service and University of Manchester approvals. Donor-matched bone marrow and subcutaneous adipose tissue was obtained from donors undergoing hip replacement surgery with full written informed consent (n = 8; average age: 59 years; age range: 29–83 years). Bone marrow aspirates were washed with phosphate buffered saline (PBS) and then centrifuged to obtain a cell pellet that was resuspended in αMEM expansion media (Sigma-Aldrich) containing 110 mg L^−1^ sodium pyruvate, 1000 mg L^−1^ glucose, 100 U mL^−1^ penicillin, 100 µg mL^−1^ streptomycin and 0.25 µg mL^−1^ amphotericin, 2 mM GlutaMAX (Life Technologies) and 10% (v/v) fetal bovine serum (FBS; expansion media). MSCs were isolated using density gradient centrifugation as previously described.^19^ Adipose tissue was dissected from non-adipose tissue, minced into small pieces and incubated at 37°C in 15 mL Hanks balanced salt solution (HBSS) containing 0.2 % (w/v) type I collagenase and 20 mM calcium chloride for 2 h with gentle agitation to allow digestion of the tissue. The digested solution was filtered through a 70 μm cell strainer, neutralised with αMEM expansion media and centrifuged for 5 min. Finally, the supernatant was aspirated, cells resuspended in expansion medium and adherent cells cultured to confluence. The CD (cluster of differentiation) profiles of donor-matched MSCs and ASCs were analysed through flow cytometry and multipotency assessed along the three mesenchymal lineages at the end of the first passage as previously reported.^20^ Cells below passage 3 were used for subsequent experiments.

### Determination of BMP receptor protein profiles

Protein was extracted from donor-matched MSCs and ASCs in culture (passage 1) using radioimmunoprecipitation assay (RIPA) buffer (50 mM Tris, pH 8.0, 150 mM NaCl, 0.1% sodium dodecyl sulfate (SDS), 5 nM ethylenediaminetetraacetic acid (EDTA), 0.5% (w/v) sodium deoxycholate, and 1% Nonidet P-40) supplemented with protease and phosphatase inhibitors as per manufacturer’s instructions (ThermoFisher Scientific) at 4°C. Insoluble material was removed from cell lysates by centrifugation (10,000 × *g*/ 10 min/4°C) and remaining protein concentration of the supernatant quantified. For western blot analysis, 20 µg total cell lysate was loaded into the wells of 4%–12% Bis/Tris Bolt gels (Life Technologies) and separated by SDS-polyacrylamide gel electrophoresis (PAGE). After electrophoresis, proteins were transferred to polyvinylidene fluoride (PVDF) membranes (ThermoFisher Scientific) and incubated with blocking buffer for 1 h at room temperature. Subsequently, membranes were incubated with primary antibodies diluted in blocking buffer containing 0.1% (v/v) Tween20 (Sigma) overnight at 4°C. Membranes were washed 5 times in tris buffered saline with 0.1 % (v/v) Tween20 (TBST). Relevant horseradish peroxidase (HRP)-conjugated secondary antibodies were incubated with membranes for 1 h at room temperature. Following incubation, membranes were washed 5 times with TBST and developed using enhanced chemiluminescent (ECL) reagent (PerkinElmer) according to the manufacturer’s instructions and exposed to photographic film. The density of each protein band was quantified using the Syngene imaging system, and the ratio of the density of bands to the density of glyceraldehyde-3-phosphate dehydrogenase (GAPDH) protein bands calculated (n = 6 donor-matched MSC and ASC populations).

The expression of BMPR2 in donor-matched MSCs and ASCs was also assessed through immunofluorescence staining analysis of BMPR2 expression in donor-matched MSCs and ASCs in monolayer culture (n = 3). To perform the staining, cells were cultured for 24 h on chamber slides in standard media to attach. Cells were then fixed in formalin, membranes permeabilized in PBS with 0.5% (v/v) Tween20. Cells were incubated with blocking buffer for 1 h at room temperature and subsequently labelled with BMPR2 primary antibody overnight at 4°C. After washing in PBS with 0.1% (v/v), Tween20 cells were incubated with alexa-488 conjugated secondary antibodies, mounted with 4′,6-diamidino-2-phenylindole (DAPI) containing mountant and imaged. The fluorescent intensity of BMPR2 staining was quantified using the CellProfiler Software developed by the Broad Institute.^21^ Briefly, an analysis pipeline was created utilising fluorescence thresholds to determine cell boundaries on immunofluorescence images and normalised for any background staining as previously described.^22^ A mask was created and superimposed over images, allowing the intensity of the fluorescent staining within defined cell areas in the mask to be calculated by the software (over 1000 MSCs and ASCs were analysed).

For analysis of BMPR2 expression between donor-matched MSC and ASC populations by flow cytometry, cells were labelled with BMPR2 antibody or isotype control primary antibody conjugated to allophycocyanin (APC) using the Lightning Link system (Innova Biosciences). Flow cytometry was performed on a BD Bioscience LSR Fortessa X-20 and labelling of cell populations compared to each other and isotype controls (n = 6).

### SMAD1, ERK1/2, and P38 MAPK phosphorylation assays

The activation of SMAD and non-SMAD pathways by rhGDF6 stimulation in culture was investigated. Cells were serum starved for 24 h prior to all experiments. Subsequently, starvation media was replaced with serum-free media containing 100 ng mL^−1^ rhGDF6 (concentration previously optimized in^10^) (PeproTech cat no. 120-04). At defined intervals, rhGDF6 containing media was removed, cells were washed twice with ice cold PBS and protein extracted in RIPA buffer or enzyme-linked immunosorbent assay (ELISA) Lysis Buffer (RayBiotech). To determine SMAD1 phosphorylation, 20 µg of cell lysate was loaded into the wells of a 4%–12% Bis/Tris Bolt gel (Thermo Fisher) and western blotting performed as previously described. Membranes were probed with phospho-SMAD1, phospho-SMAD2, pan-SMAD1, and pan-SMAD2 antibodies (n = 3). Protein band intensities were quantified as described above. Additionally, a phospho-SMAD1 ELISA (RayBiotech) was performed according to manufacturer’s instructions and SMAD1 phosphorylation quantified through measuring absorbance at 450 nm (n = 3). Activation of ERK1/2 and P38 MAPK pathways by rhGDF6 stimulation was investigated through western blot. Protein extraction and western blots were performed as above and membranes probed with phospho- and pan-ERK1/2 and P38 MAPK antibodies (Cell Signalling Technology) (n = 3). Protein band intensities were quantified as above. Details of all primary and secondary antibodies used in this study can be found in Supplementary Table 1.

### ASC Differentiation to a NP-like phenotype in 3D culture

To investigate rhGDF6-mediated NP-like differentiation of ASCs, cells were cultured in 3D NP-inductive conditions for 2 weeks with and without additional rhGDF6 stimulation at 100 ng mL^−1^.^10^ ASCs were loaded at high density (4.0 × 10^6^ cells mL^−1^) into type I collagen gels (Devro; 3 mg mL^−1^) and 100 µL placed into 0.4-µm high-density cell culture inserts in 24-well plates. After gels were set, they were stabilized for 24 h in expansion media after which media was changed for NP-inductive media (AQmedia high-glucose Dulbecco’s Modified Eagle Medium (DMEM), 1% fetal calf serum (FCS), insulin-transferrin-selenium (ITS-X), 100 µM ascorbic acid-2-phosphate, 1.25 mg mL^−1^ bovine serum albumin, 10^−7^M dexamethasone, 5.4 µg mL^−1^ linoleic acid, 40 µg mL^−1^ L-proline, 100 U mL^−1^ penicillin, 100 µg mL^−1^ streptomycin and 0.25 µg mL^−1^ amphotericin) with or without addition of 100 ng mL^−1^ rhGDF6.^10^

The role of specific pathways in rhGDF6-mediated differentiation was investigated by selective inhibition of SMAD1/5/8 and ERK1/2 using the small molecule inhibitors dorsomorphin (10 µM) and U0126 (10 µM), respectively. Pathways were blocked prior to gel formation with pre-incubation with inhibitors and inhibitors replenished at every media change. Inhibition was confirmed through protein analysis of pathway phosphorylation either through phospho-SMAD1 ELISA or phospho-ERK1/2 western blot. After 2 weeks culture, either RNA was extracted for qPCR analysis or gels were immunostained for NP-like ECM markers.

### Assessment of NP-like differentiation – qPCR and immunohistochemical staining

Expression of NP marker genes *SOX9, ACAN, COL2A1, KRT8, KRT18, KRT19*, and *FOXF1* was assessed through qPCR after 14 days, as previously described.^10^ Briefly, biological samples were investigated in triplicate and 2 µL of cDNA (5 ng/µL) was pipetted into each reaction well. A positive and negative sample was run for each gene examined to ensure no false positives; total human RNA and molecular grade water replaced cDNA, respectively. Data were analysed according to the 2^−ΔCt^ method and noramlised to two internal, prevalidated reference genes, MRPL19 and GAPDH. Optimised primer/probe sequences for the genes utilised throughout this study are detailed in Supplementary Table 2. Aggrecan and type II collagen production was investigated through immunohistochemical (IHC) analysis of gels. Briefly, gels were embedded into OCT and frozen on dry ice, cut into 8-µm-thick cross-sections and collected onto SuperFrost Plus slides. Sections were allowed to equilibrate to room temperature, formalin fixed and blocked prior to addition of either aggrecan (Bio-Rad; MCA1454G) or type II collagen (Proteintech 15943-1-AP) primary antibodies and incubated at 4°C overnight. Relevant biotinylated secondary antibodies (Vector labs BA-1000 or BA-9200) were added after washing (5 × 5 min TBS with 0.1 % (v/v) Tween 20; TBST) and incubated at room temperature for 1 h. Following washing with TBST, specific staining was visualized through incubation at room temperature with DAB Chromagen.

### Statistical Analysis

To compare receptor expression between MSCs and ASCs and signalling pathway phosphorylation experiments non-parametric Mann-Whitney *U* tests were applied to determine significance; values are reported and mean and SEM values shown graphically. For flow cytometry BMPR2 expression (fluorescence intensity (APC)) in donor-matched MSC and ASC populations was compared using Kolmogorov-Smirnov (K-S) tests and mean population intensities were compared through T-tests. BMPR2 expression was also compared through quantification of BMPR2 immunofluorescently stained cells and statistical significantly differences between cell populations determined by Mann-Whitney *U* tests. Analysis of differences between groups using qPCR data was conducted through one-way analysis of variance (ANOVA) analysis including Dunnett’s multiple comparisons test comparing all groups to control group, which was GDF6 stimulated samples or no stimulation samples depending on the analysis. Values shown are means ±SEM. For all analyses, a value of p < 0.05 was considered statistically significant. All analyses were conducted using GraphPad Prism 7 Software.

## Results

### BMPR2, a type II GDF6 receptor, is differentially expressed in MSCs and ASCs

**Figure 1.**
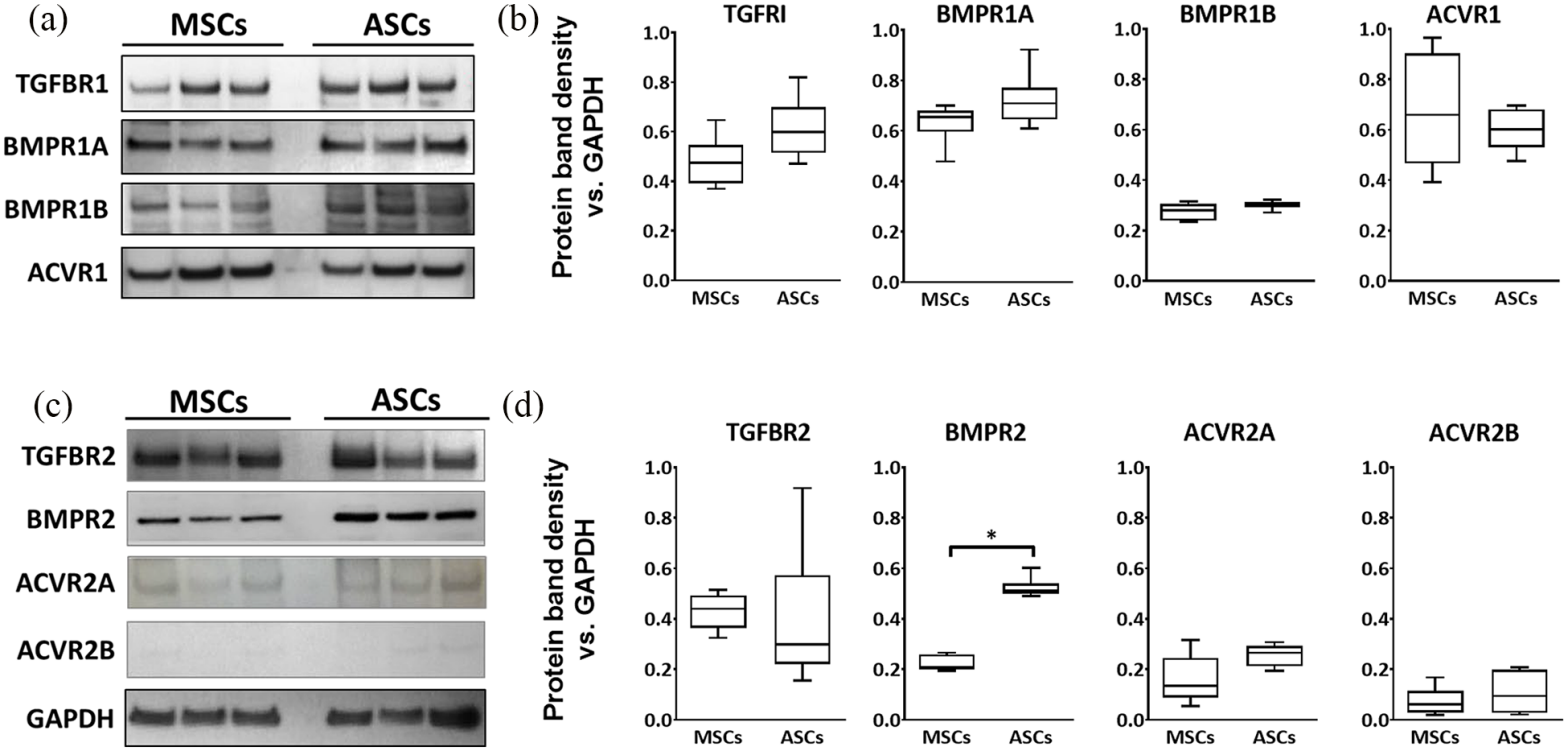
(a) Western blot images and (b) densitometric analysis comparing the expression of type I TGF/BMP receptors between donor-matched MSCs and ASCs. (c) Western blot images and (d) densitometric analysis comparing type II TGF/BMP receptors between donor-matched MSCs and ASCs. GAPDH expression in samples was used an internal loading control. Blots show representative comparisons for three donor-matched populations. Densitometric data show mean values ± SEM. (n = 6; *p < 0.05). Receptor profiles for BMP receptors showed similar expression for MSCs and ASCs, with the exception of BMPR2, which was significantly higher in ASCs.

As a BMP family member, the cell surface receptors for rhGDF6 are composed of heteromeric complexes containing type I and type II receptor subunits. As such, type I and type II BMP receptor subunit protein expression was profiled in donor-matched MSCs and ASCs (n = 6) through western blot (Figure 1). rhGDF6 is known to have the ability to bind to the type I receptors BMPR1A and BMPR1B. Here BMPR1A was strongly expressed by both MSCs and ASCs while BMPR1B expression appeared weaker (Figure 1a). Though no statistically significant difference in expression of either receptor was observed between the two populations, there was a trend for stronger expression of BMPR1A in ASCs (Figure 1b). Importantly, analysis of type II receptor subunit expression between MSCs and ASCs (Figure 1c) revealed significantly higher expression of BMPR2 in ASC populations compared to donor-matched MSCs (p = 0.0022) (Figure 1d). The expression of ACVR2A, a hypothesized GDF6 receptor, appeared higher in ASCs than MSCs, although the level of expression was appreciably lower in both populations compared to other type II receptors and variable leading to no statistically significant difference.

**Figure 2.**
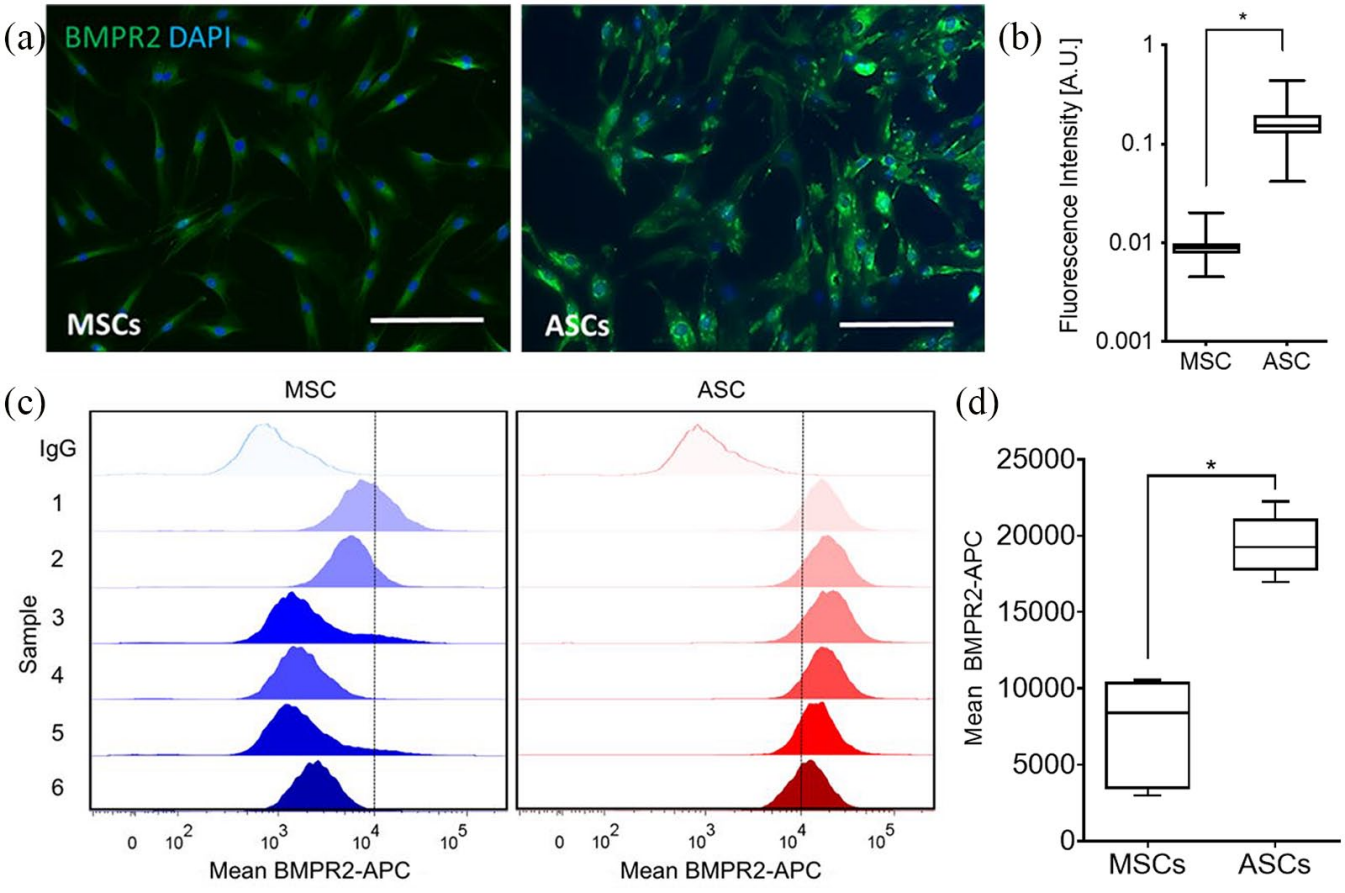
(a) Representative immunofluorescence images and (b) quantification of fluorescence intensity (CellProfiler Software) comparing BMPR2 (Alexa488) expression between MSCs and ASCs *in vitro* (n = 3; *p < 0.001). Nuclei stained with DAPI. Scale bars = 200 µm. Expression of BMPR2 was observed to be greater in ASCs compared to MSCs. (c) Flow cytometric analysis of donor-matched MSC and ASC populations stained with BMPR2-APC. One representative isotype control is shown for each cell type for clarity. (d) Mean population fluorescent intensity shown graphically; ASCs showed significantly higher staining intensity for BMPR2 in comparison to MSCs, with less variation in mean expression observed (n = 6; *p < 0.01).

ICC analysis (Figure 2a, 2b) and flow cytometry (Figure 2c, 2d) targeting BMPR2 were also performed to confirm differences between donor-matched MSC and ASC populations. Immunofluorescence of donor-matched populations showed a greater intensity of BMPR2 staining for ASCs, which when quantified was shown to be statistically significantly higher in comparison to MSCs (p < 0.001). Flow cytometric analysis of donor-matched MSC and ASC populations further demonstrated differences in BMPR2 expression. Both MSCs and ASCs were found to be positive for BMPR2, consistent with western blot and immunofluorescence analysis. In further agreement with these other analyses, when BMPR2-APC stained populations were compared, ASCs were found to have statistically significant increases in BMPR2 staining (p = 0.0012), while the mean fluorescence intensities of ASC populations were found to be less variable, indicating less inter-donor variation in GDF6-receptor expression.

### rhGDF6 activation of both SMAD1/5/8 and non-SMAD pathways is greater in ASCs in comparison to MSCs

**Figure 3.**
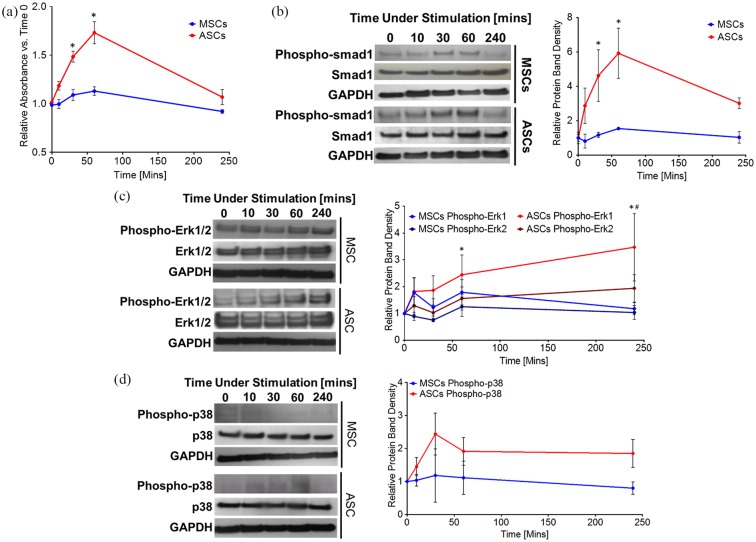
(a) Phospho-SMAD1 ELISA comparing donor-matched MSCs and ASCs over a 4-h rhGDF6 stimulation time-course. MSCs and ASCs were serum starved and then stimulated with serum-free media containing 100 ng mL^−1^ rhGDF6. Data shown are mean values ± SEM. (n = 3 in independent triplicates; *p < 0.05). (b) Representative western blot images and (c) densitometric quantification of phospho-SMAD1 expression in donor-matched MSC and ASC populations over a 4-h rhGDF6 stimulation time-course. Quantification data shown are mean values ± SEM. (n = 3; *p < 0.05). (d) Representative western blot images of ERK1/2 and P38 phosphorylation following stimulation with 100 ng mL^−1^ rhGDF6 in donor-matched MSCs and ASCs over a 4-hour rhGDF6 stimulation time-course (e) Densitometric analysis of western blot of phosphorylation of ERK1/2 and P38 following stimulation with 100 ng mL^−1^ rhGDF6 in donor-matched MSCs and ASCs over a 4-hr rhGDF6 stimulation time-course. Data shown are mean values ± SEM (n = 3; *p < 0.05 ERK1 ASC vs. ERK1 MSC; #p < 0.05 ERK2 ASC vs. ERK2 MSC).

Following the finding that BMPR2 receptor expression was higher in ASCs compared to MSCs, we aimed to determine if a greater expression of rhGDF6 receptors correlated with a greater activation of canonical (SMAD) and non-canonical (ERK1/2 or P38) pathways downstream of receptors in response to rhGDF6 stimulation in ASCs compared to MSCs. As a BMP family member, rhGDF6 is thought to act through the SMAD1/5/8 pathway and, in agreement with this, no SMAD2/3 activation by rhGDF6 stimulation was identified in either MSCs or ASCs, as determined by phospho-SMAD2 western blot analysis (Supplementary Figure 1). Following rhGDF6 stimulation in culture SMAD1 phosphorylation was determined by phospho-SMAD1 ELISA (Figure 3a) and western blot (Figure 3b). The phosphorylation of SMAD1 was found to be increased in ASCs in comparison to MSCs after rhGDF6 stimulation determined by both ELISA (30 min p = 0.008; 60 min p = 0.022) and quantification of western blot analysis (60 min p = 0.044; 240 min p = 0.012). SMAD1 phosphorylation also increased more rapidly in ASCs than in MSCs.

Some BMP family members have also been shown to activate non-SMAD signalling pathways, and it was hypothesized that rhGDF6 may also be able to activate non-SMAD kinase cascades, specifically ERK1/2 and P38 MAPK. Following rhGDF6 stimulation, ERK1/2 was strongly phosphorylated above baseline levels in ASCs (Figure 3c). Similar to SMAD1 phosphorylation, ERK1/2 phosphorylation in ASCs was seen to be significantly higher than in donor-matched MSCs (p-ERK1 60 min stimulation p = 0.0059, 240 min stimulation p < 0.001; p-ERK2- 240 min stimulation p = 0.0023). Temporally, the activation of these pathways showed a different profile to SMAD1, with greatest activation seen at 240 min post-rhGDF6 stimulation. P38 MAPK was not significantly induced by rhGDF6 stimulation in either cell population, indicating that this pathway is not directly involved in cell response to rhGDF6 (Figure 3d).

### rhGDF6 stimulation promotes a discogenic phenotype in ASCs in 3D culture that is attenuated by SMAD1/5/8 and ERK1/2 inhibition

**Figure 4.**
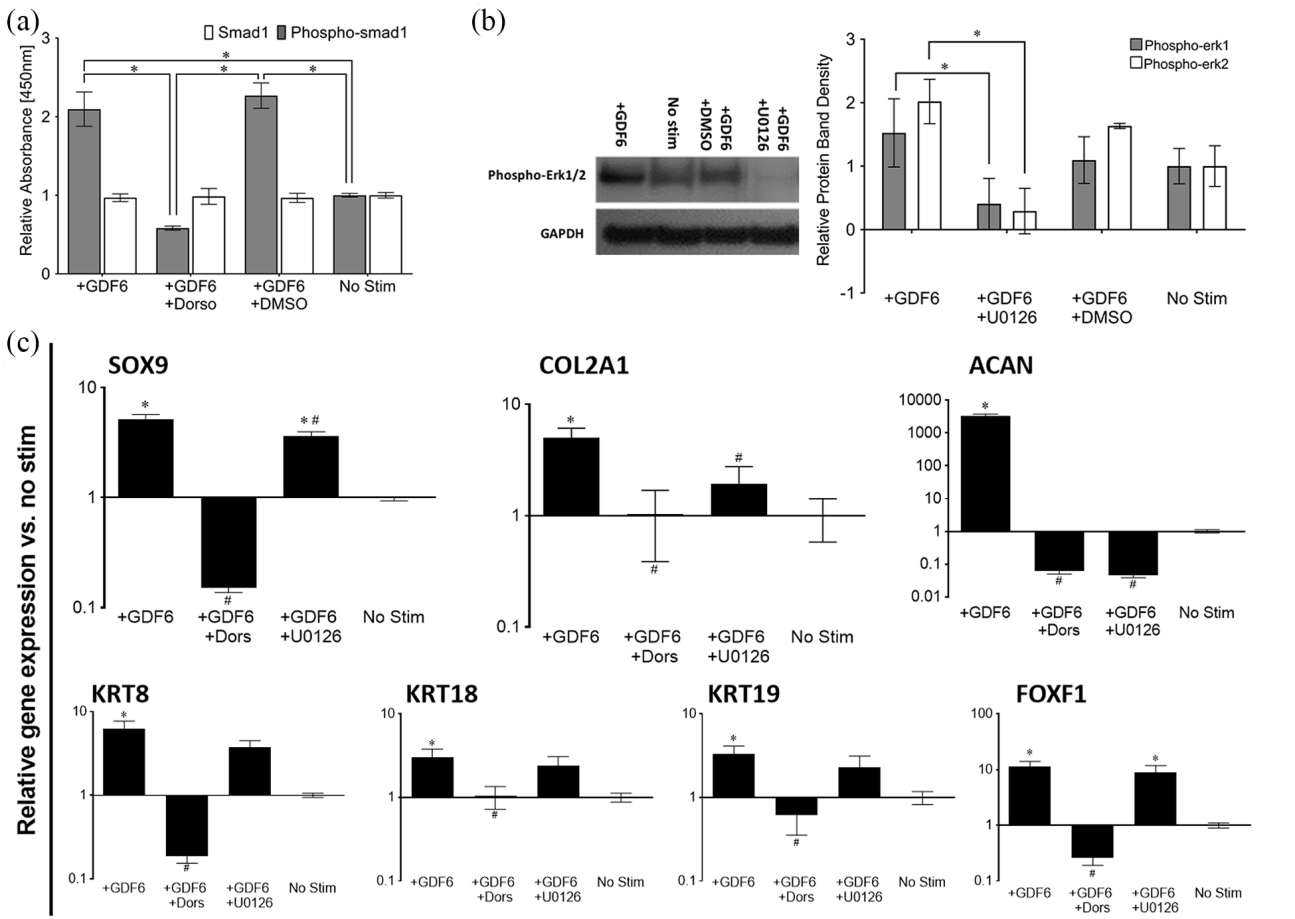
(a) Phospho-SMAD1 ELISA analysis of dorsomorphin (Dorso) blocking of rhGDF6-mediated SMAD1 phosphorylation following stimulation with 100 ng mL^−1^ rhGDF6. Data shown are mean values ± SEM (n = 3; *p < 0.05). Phospho-SMAD1 ELISA shows rhGDF6-induced phosphorylation of SMAD1 after 60 min stimulation with 100 ng mL^−1^ rhGDF6, which is returned to control levels with addition of 10 µM dorsomorphin. (b) Representative western blot analysis showing effective blocking of ERK1/2 phosphorylation in ASCs by culture with 10 µM U0126 and densitometric analysis of western blots of ERK1/2 phosphorylation inhibition in ASCs following 100 ng mL^−1^ rhGDF6 stimulation. Data shown are mean values ± SEM. (n = 2; *p < 0.05). rhGDF6 effectively induces ERK1/2 phosphorylation after 60 min culture with 100 ng mL^−1^ rhGDF6, which is abrogated by culture with U0126. (c) Quantitative real-time PCR analysis of healthy NP-marker gene expression in ASCs after 14 days of 3D collagen gel culture with 100 ng mL^−1^ rhGDF6 stimulation with and without SMAD1/5/8 inhibition (dorso) or ERK1/2 inhibition (U0126). Relative gene expression was normalized to mean housekeeping gene expression and fold change calculated vs. no stimulation control cells (n = 3 donor populations in triplicate; data represents mean ± SEM; *p < 0.05 vs rhGDF6 stimulated cells).

To determine the relative effects of each pathway on NP-like differentiation of ASCs, SMAD1 and ERK1/2 pathway activation were specifically blocked through small molecule inhibitors. Inhibition of SMAD1/5/8 and ERK1/2 signalling was confirmed in each case, as expected (Figure 4a, 4b). ASCs were then cultured for 14 days in 3D collagen gel cultures under conditions known to induce NP-like differentiation,^10^ with inhibitors added at the start of culture and again at each media change. rhGDF6 significantly upregulated expression of all NP marker genes in ASCs in 3D collagen gel culture compared to cells cultured for 14 days in the absence of rhGDF6 (no stimulation) (p < 0.001) (Figure 4c). This effect was abrogated completely by inhibition of SMAD1/5/8 by dorsomorphin, indicating that SMAD activation is required for rhGDF6-mediated induction of NP-marker genes in ASCs. Indeed, expression decreased to levels below baseline, indicating a role for SMAD signalling in maintenance levels of expression of these genes. Furthermore, inhibition of ERK1/2 activation by rhGDF6 showed a general trend resulting in decreased NP-marker gene expression with significant decreases in *SOX9* (p = 0.0055), a critical NP cell transcription factor required for type II collagen and aggrecan expression, *COL2A1* (p = 0.042), *ACAN* (p < 0.0001) and keratin 8 (p = 0.008). Of importance to NP regeneration, *ACAN* was the most responsive gene to rhGDF6 stimulation, increasing dramatically in comparison to unstimulated ASCs. This increased expression relied on both SMAD1/5/8 and ERK1/2 signalling (p < 0.0001).

**Figure 5.**
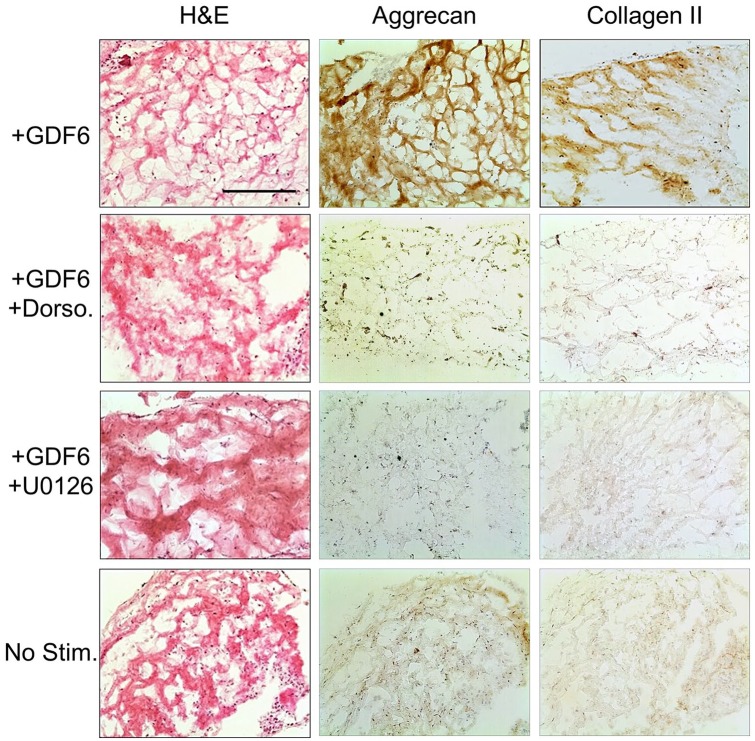
Immunohistochemical analysis of ASC collagen gel cultures after 14 days with 100 ng mL^−1^ rhGDF6 stimulation with and without SMAD1/5/8 (Dorso) or ERK1/2 (U0126) inhibition and no stimulation control. After 14 days culture, constructs stimulated with rhGDF6 demonstrated an increase in aggrecan and type II collagen deposition compared to rhGDF6 stimulated constructs where SMAD1/5/8 and ERK1/2 signalling had been blocked, where levels appeared similar to unstimulated controls. Scale bar, 500 µm.

These results were mirrored at the protein level as assessed IHC analysis of aggrecan and type II collagen in ASC gel cultures at 14 days (Figure 5). rhGDF6 stimulation significantly increased the amounts of aggrecan and type II collagen secreted by cultured cells. This production was returned to levels comparable to unstimulated control levels by culture with SMAD1/5/8 inhibition. Selective inhibition of ERK1/2 also significantly decreased aggrecan and type II collagen production, though levels remained marginally increased from unstimulated controls, a finding in close parallel to gene expression data.

## Discussion

GDF6 is required for the correct development of the IVD in mice and humans and is expressed in adult IVD tissues.^8,20,23-25^ The delivery of rhGDF6 in combination with stem cells to degenerative IVDs holds promise as a regenerative strategy but previous reports have highlighted key differences in MSC and ASC response to rhGDF6. Here, we aimed to investigate the mechanism of rhGDF6 induction of NP-like phenotypes in MSCs and ASCs through examination of receptor expression, signalling pathway activation, pathway specific blocking and the phenotypes that resulted.

GDF6 receptor profiles in donor-matched MSCs and ASCs demonstrated that while type I receptor profiles were similar between populations, ASCs demonstrated a significantly higher expression of BMPR2, a type II receptor subunit known to bind to GDF6, compared to MSCs. Correspondingly, with rhGDF6 stimulation *in vitro* ASCs had greater increases in SMAD1 and ERK1/2 phosphorylation than MSCs. This finding provides strong evidence that previously observed differences in rhGDF6-mediated increases in NP-marker gene expression between MSCs and ASCs^10^ could be due to differing levels of signal transduction, in part due to variation in receptor availability and resultant signalling. This variation reinforces that expanded MSC and ASC populations are not identical and respond differently to rhGDF6 in terms of differentiation to an NP phenotype.

Though to date we are the only group to have directly examined the comparative differentiation of donor-matched MSCs and ASCs into NP-like cells,^10^ previous reports on the different differentiation capacities of MSCs and ASCs along chondrogenic lineages serve to highlight the importance of receptor expression profile on cell responses.^14-18^ Hennig and co-workers report that ASCs have a reduced capacity for *in vitro* chondrogenic differentiation, a finding also described by other researchers.^14,15,26^ However, several important differences between these previous studies and the results described in the present study may account for this apparent discrepancy. Critically, chondrogenic differentiation in these previous works relies on TGFβ media supplementation rather than GDF6, which acts through alternative cell surface receptors and downstream SMAD signalling pathways. Importantly, increased TGF signalling has been shown to produce suboptimal phenotypes for NP engineering.^27^ In one study, the differences between MSCs and ASC responses were ascribed to absence of TGFBR1/ALK-5 gene expression in ASCs, that was reversible through supplementation with BMP6 which eliminated chondrogenic differentiation deficit.^14^ We found no significant difference in TGFBR1/ALK-5 expression at the protein level between MSCs and ASCs, with high expression found in both – a finding supported by previous work.^28^ Here, NP-like differentiation is induced by supplementation with rhGDF6, which does not use TGFBR1 or SMAD2/3 signalling. This may indicate that despite a heightened response to TGFβ family signalling (shown to be sub-optimal for NP differentiation) in MSCs, it is ASCs that are more responsive to GDF family members, which provide vital cues to synthesise the specialized ECM of the NP. Though the end-point phenotype of NP cells shows some similarities to that of chondrocytes, it may be that the transcriptional programs produced by these differing signalling mechanisms are, critically, sufficiently divergent to create a functional NP ECM. Importantly, for the potential therapeutic use of these cell populations, flow cytometry indicates less intrapopulation variation in BMPR2 expression in ASCs compared to MSCs. The implication of this is that the selection of ASCs over MSCs may be key for achieving reproducible responses from cells implanted *in vivo*.

Through selective blocking of candidate intracellular signalling pathways we identified that both SMAD1/5/8 and ERK1/2 have importance in the translation of rhGDF6 signalling into gene expression. Aggrecan and type II collagen are key components of the healthy NP ECM that are downregulated during degeneration. Here, rhGDF6 stimulation significantly induced type II collagen and aggrecan expression but importantly resulted in a greater induction of aggrecan than collagen, which is more indicative of an NP phenotype. Of particular interest to NP engineering was the finding that both SMAD1/5/8 and ERK1/2 are required for observed increases in aggrecan expression and that blocking either pathway significantly decreased aggrecan production at both gene and proteoglycan levels. Blocking ERK1/2 significantly decreased *SOX9* expression, though not to the extent of blocking SMAD1/5/8. This indicates that ERK1/2 signalling exerts its effects on aggrecan expression through interaction with other cell processes, although more work is required to elucidate these signalling pathways.

In addition to aggrecan and type II collagen, SMAD1/5/8 signalling was required for the upregulation of all NP-specific marker genes. The importance of this pathway in healthy NP-like gene expression is perhaps unsurprising and upregulation of the inhibitory SMAD-7 has been positively correlated with increasing IVD degeneration and is highly expressed in severe degeneration.^29^ Meanwhile SMAD-6, an inhibitory SMAD that along with SMAD-7 blocks BMP/TGFβ signalling, has been strongly linked to degenerative changes occurring in the NP with aging.^30^ rhGDF6 stimulation with the implantation of healthy cells, as investigated here, may allow rebalancing of this anabolic/catabolic equilibrium.

Interestingly, we also found that ERK1/2 signalling was important for the full induction of NP-specific genes by GDF6, with blocked cells displaying an attenuated expression of NP-specific markers. As other BMP-family members are able to signal through other mitogen-activated protein kinase (MAPKs),^31^ we investigated P38 MAPK activation by rhGDF6 but did not observe significant phosphorylation following rhGDF6 stimulation. This result corresponds with previous work on the close family member GDF5, which found ERK1/2 but not P38 activation.^32^ ERK1/2 signalling has been previously linked to establishment and maintenance of NP phenotypes in organ-culture models^33^ and ERK1/2 activation has been linked to cell culture in hypoxic and hypertonic environments,^34,35^ such as experienced in the NP. This suggests that ERK1/2 activation is involved in environmental adaptation of NP cells, inducing NP-like gene expression and that rhGDF6 is involved in stimulating or enhancing this effect.

The results reported here have implications for the development of stem cell therapies for disc degeneration and are a step towards optimization of rhGDF6-mediated treatments. The robust and repeatable response of ASCs to rhGDF6 stimulation in 3D culture reinforce previous results from our laboratory and suggest that as a therapeutic biologic rhGDF6 may currently be the most promising candidate for differentiation of ASCs to and maintenance of NP-like cells. The determination that BMPR2 expression correlates accurately with increased downstream signalling responses and that ASCs express higher levels of BMPR2 is likely to be important information to maximize the efficacy of cell therapies.

## Conclusion

In conclusion, we have identified key underlying differences in both GDF6 receptor subunit expression and downstream signal transduction between donor-matched MSCs and ASCs that correlate with increased responses observed here and previously^10^ to rhGDF6 stimulation in ASCs. SMAD1/5/8 signalling was found to be required for GDF6-induced NP-marker gene expression. For the first time, rhGDF6 was also found to activate ERK1/2 signalling and blocking of pathway phosphorylation attenuated NP marker gene expression. The data presented here will aid in the selection of the most efficacious cell populations for IVD regeneration and the optimization of their therapeutic effect.

## Supplemental Material

SI_High_BMPR2_expression_leads_to_enhanced_smad1 – Supplemental material for High BMPR2 expression leads to enhanced SMAD1/5/8 signalling and GDF6 responsiveness in human adipose-derived stem cells: implications for stem cell therapies for intervertebral disc degeneration Click here for additional data file. Supplemental material, SI_High_BMPR2_expression_leads_to_enhanced_smad1 for High BMPR2 expression leads to enhanced SMAD1/5/8 signalling and GDF6 responsiveness in human adipose-derived stem cells: implications for stem cell therapies for intervertebral disc degeneration by Tom Hodgkinson, Francis Wignall, Judith A Hoyland and Stephen M Richardson in Journal of Tissue Engineering
