# Supplementary material for: High BMPR2 expression leads to enhanced SMAD1/5/8 signalling and GDF6 responsiveness in human adipose-derived stem cells: implications for stem cell therapies for intervertebral disc degeneration
Source: J Tissue Eng. 2020 May 18;11:2041731420919334. doi: 10.1177/2041731420919334 (PMC7238299; doi:10.1177/2041731420919334)

High BMPR2 expression leads to enhanced SMAD1/5/8 signalling and GDF6 responsiveness in human adipose-derived stem cells: implications for stem cell therapies for intervertebral disc degeneration

Tom Hodgkinson[1], Francis Wignall[1], Judith A Hoyland[1,2], Stephen M. Richardson[1]

[1] Division of Cell Matrix Biology and Regenerative Medicine, School of Biological Sciences, Faculty of Biology, Medicine and Health, University of Manchester, Manchester Academic Health Science Centre, Oxford Road, Manchester M13 9PT, UK.

[2] NIHR Manchester Musculoskeletal Biomedical Research Unit, Central Manchester Foundation Trust, Manchester Academic Health Science Centre, Manchester, UK

Supplementary Information

Corresponding Author:

Dr. Stephen M. Richardson

Division of Cell Matrix Biology and Regenerative Medicine, School of Biological Sciences, Faculty of Biology, Medicine and Health, The University of Manchester, Manchester Academic Health Science Centre, Oxford Road, Manchester M13 9PT, UK

Email: S.Richardson@manchester.ac.uk

Supplementary Table 1

Target Protein	Company	Catalogue Number	Dilution
**Type I Receptor**			
TGFRI	Abcam	Ab31013	1:500
BMPR1A	Millipore	MABS419	1:500
BMPR1B	Abcam	Ab175385	1:500
ACVRI	Abcam	Ab155981	1:1000
**Type 2 Receptors**			
TGFBR2	Abcam	Ab61213	1:500
BMPR2	Thermo Scientific	MA5-15827	1:1000 (WB)<br>1:100 (IF)<br>1:1000 (Flow)
ACVR2A	Abcam	Ab134082	1:500
ACVR2B	Abcam	Ab180185	1:1000
**Signalling pathways**			
P-SMAD1/5/9	Cell Signalling Technology	13820	1:1000
SMAD1	Cell Signalling Technology	6944	1:1000
P-SMAD2	Cell Signalling Technology	3108	1:1000
SMAD2	Cell Signalling Technology	5339	1:1000
P-ERK1/2	Cell Signalling Technology	4377	1:1000
ERK1/2	Cell Signalling Technology	4695	1:1000
P-P38	Cell Signalling Technology	9215	1:1000
P38	Cell Signalling Technology	9212	1:1000
P-MAPKAPK-2	Cell Signalling Technology	3316	1:1000
**Secondaries**			
Anti-mouse IgG (Goat)-HRP	Perkin Elmer	NEF822001EA	W.B. 1:10000
Anti-rabbit IgG (Goat)-HRP	Perkin Elmer	NEF812001EA	W.B. 1:10000
Anti-mouse (Goat)-AF488	Thermo Scientific	A-11029	IF 1:300

Supplementary Table 2

Gene	Forward Primer	Reverse Primer
SOX9	GACTTCCGCGACGTGGAC	CAGTACCTGCCGCCCAAC
ACAN	TCGAGGACAGCGAGGCC	TCGAGGGTGTAGCGTGTAGAGA
COL2A1	GGCAATAGCAGGTTCACGTACA	CGATAACAHTCTTGCCCCACTT
KRT8	TGACCGACGAGATCAACTTCCT	TGGACAGCACCACAGATGTGT
KRT18	GCGAGGACTTTAATCTTGGTGATG	TGGTCTTTTGGATGGTTTGCA
KRT19	GGTCATGGCCGAGCAGAA	TTCAGTCCGGCTGGTGAAC
FOXF1	GCCGTATCTGCACCAGAACA	CGTTGAAAGAGAAGACAAACTCCTT
GAPDH	ATGGGGAAGGTGAAGGTCG	TAAAAGCAGCCCTGGTGACC
MRPL19	CACCGCCCCGTGGAA	TCCCCTTCGAGGAATGAATTC

Supplementary Figure 1: rhGDF6 does not phosphorylate SMAD2 in culture. Donor-matched MSCs and ASCs were serum starved for 16 hours and subsequently stimulated with **(A)** 100 ng ml^{-1} rhGDF6 or **(B)** 10 mg ml^{-1} TGFβ3 in serum-free media. Phosphorylation of SMAD2 was assessed by western blot after protein extraction at defined time points. Phospho-SMAD2 was activated by TGFβ3 but not rhGDF6

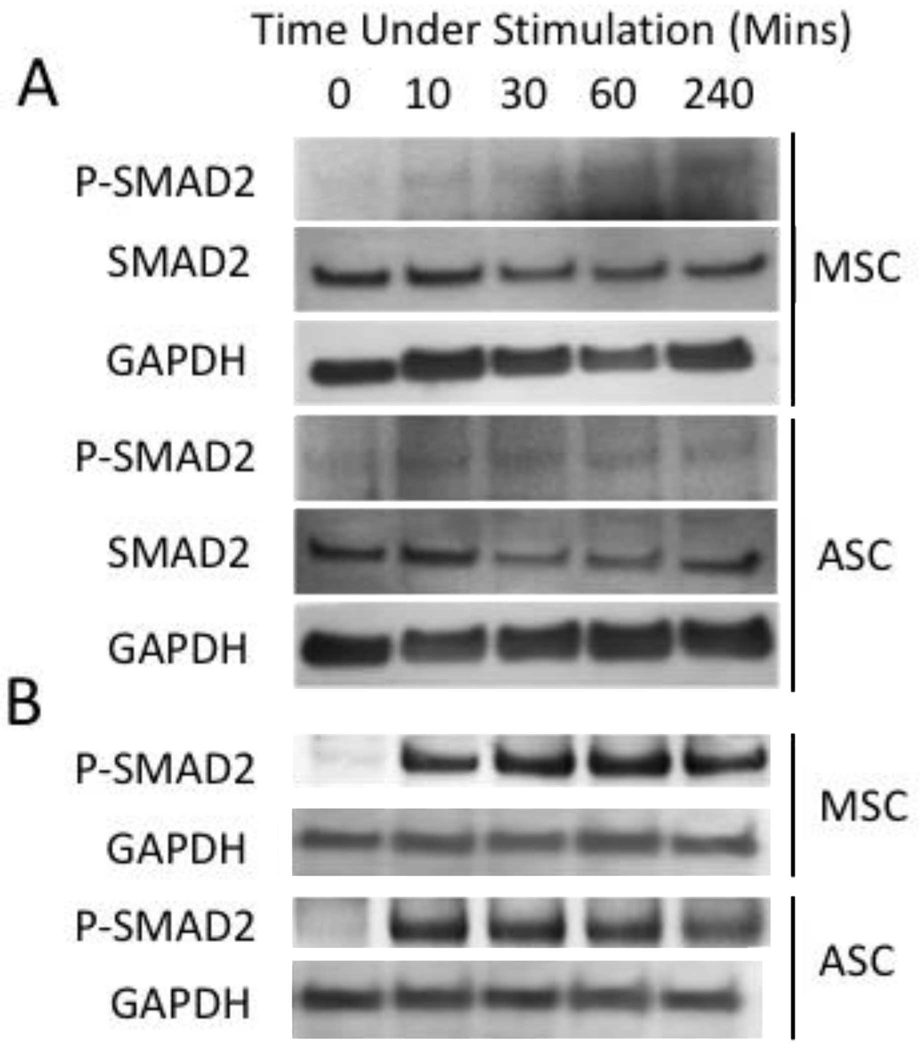

Supplement: SI_High_BMPR2_expression_leads_to_enhanced_smad1 – Supplemental material for High BMPR2 expression leads to enhanced SMAD1/5/8 signalling and GDF6 responsiveness in human adipose-derived stem cells: implications for stem cell therapies for intervertebral disc degeneration [file SI_High_BMPR2_expression_leads_to_enhanced_smad1.pdf]
